# Quantitative distribution of essential elements and non-essential metals in breast cancer tissues by LA-ICP-TOF–MS

**DOI:** 10.1007/s00216-024-05652-8

**Published:** 2024-11-18

**Authors:** Sara Escudero-Cernuda, David Clases, Noemi Eiro, Luis O. González, María Fraile, Francisco J. Vizoso, María Luisa Fernández-Sánchez, Raquel Gonzalez de Vega

**Affiliations:** 1 Department of Physical and Analytical Chemistry, University of Oviedo, Oviedo, Spain; 2 Institute of Chemistry, University of Graz, Graz, Austria; 3 Research Unit, Jove Hospital Foundation, Gijón, Spain

**Keywords:** Elemental bioimaging, Elemental quantification, Hyphenated techniques, LA-ICP-MS, Time of flight

## Abstract

Breast cancer (BC) is the leading cause of cancer death among women worldwide, making the discovery and quantification of new biomarkers essential for improving diagnostic and preventive strategies to limit dissemination and improve prognosis. Essential trace metals such as Fe, Cu, and Zn may play critical roles in the pathophysiology of both benign and malignant breast tumors. However, due to the high metabolic activity and reduced element selectivity of cancer cells, also non-essential elements may be taken up and may even be implicated with disease progression. This study investigates the spatial distribution and concentrations of both essential and non-essential elements in breast tissues, assessing their potential for diagnostic applications. Laser ablation (LA)–inductively coupled plasma–mass spectrometry (ICP-MS) with a time-of-flight (ToF) mass analyzer (LA-ICP-ToF–MS) was used to inquire the distribution of almost all elements across the periodic table and their abundance in metastatic (*n* = 11), non-metastatic (*n* = 7), and healthy (*n* = 4) breast tissues. Quantification was achieved using gelatine-based standards for external calibration to quantitatively map various elements. Overall, the Fe, Cu, Zn, Sr, and Ba levels were significantly increased in tumor samples with Sr and Ba showing strong correlation, likely due to their similar chemistry. Comparison of calibrated LA-ICP-ToF–MS data with a histologic staining demonstrated the possibility to clearly differentiate between various tissue types and structures in breast tissues such as tumor niche and stroma. The levels of the studied elements were significantly higher in the tumor niche areas compared to the stroma, and for Fe, a significant accumulation was observed in the tumor niche areas from the metastatic patient group relative to the levels found in the same areas of the non-metastatic group.

**Graphical Abstract:**

LA-ICP-ToF–MS was used to quantitatively map the biodistribution of essential and non-essential elements in metastatic and non-metastatic breast cancer tissues.

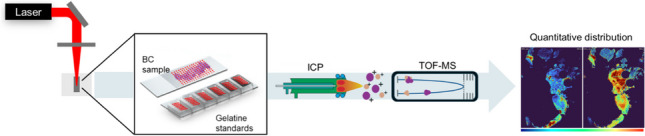

**Supplementary Information:**

The online version contains supplementary material available at 10.1007/s00216-024-05652-8.

## Introduction

Breast cancer (BC) is the leading cause of cancer death among women worldwide [1, 2], and a better understanding of metabolic alterations related to this disease is required to identify and exploit new biomarkers for early detection, diagnosis, prognosis, and treatment [3]. Essential trace elements like iron (Fe), copper (Cu), and zinc (Zn) have relevant functions in many cellular processes and are cofactors for countless proteins associated with cancer including development, suppression, progression and metastasis [4, 5]. While Fe and Cu are, for example, bound to metalloproteins that are involved in several physiological processes like mitochondrial respiration, biosynthesis of hormones, and antioxidant defense [6, 7], Zn plays crucial roles in cell growth and development, metabolism, and immune system function [8]. Zn is also cofactor of proteins correlated with cancer invasion like the matrix metalloproteinases (MMPs) [9–11].

It is well studied that accumulation or deficiency of these elements can cause cell damage, DNA damage, and oxidative stress, both of which can lead to the development of a variety of cancers. For example, deficiency in essential metals, such as Zn, Fe, and selenium (Se), were reported to be associated with increased risks of esophageal, gastric, and colon cancers [12–15], and changes in the distribution of trace elements in tissues and serum have been reported in patients with various types of cancers [16–18]. Despite extensive investigations into Fe, Cu, and Zn concentrations in the serum and tissues of cancer patients, their precise roles in tumorigenesis remain unclear. Hua et al. suggested that serum Fe level may have a diagnostic utility for patients with early stage of triple negative BC [19]. Feng et al. found that elevated Cu levels and decreased Zn levels in serum are associated with risk of breast cancer [20]. These studies provide evidence for the relationships between the regulation of various elements and their potential implication in the pathogenesis of BC.

On the other hand, several studies suggested that heavy metals such as cadmium (Cd), chromium (Cr), lead (Pb), mercury (Hg), and nickel (Ni) may be implicated in tumorigenesis and act as potential carcinogens increasing the susceptibility to tumor development and progression in humans [21–24]. However, the research on the associations between cancer and the exposure to many elements such as strontium (Sr) and barium (Ba), which will be a focus of this study, is still limited. Sr, like calcium (Ca), enhances the extracellular ERK/MAPK signaling pathway [25] that plays a central role in the signal transduction network promoting tumor initiation and progression [26]. Also, Sr has been detected by LA-ICP-ToF–MS in human malignant mesothelioma tissues and correlated with asbestos fibers [27]. Nowadays, there is little evidence about the carcinogenic toxicity of Ba; nevertheless, an in vitro study showed that exposition to Ba for a short time promotes the transforming activity of various non-tumorigenic cells [28]. These results suggest that Sr and Ba may be implicated in the development of cancer, highlighting the need for further studies on their exposure, accumulation behavior, and their physiological and pathological pathways. Recent studies investigating the interaction between cancer cells and the surrounding stroma, which is significantly responsible for tumor growth and metastasis, support the importance of understanding such elements in the tumor microenvironment. Characteristic stroma alterations accompany or even precede the malignant conversion of epithelial cells [29]. One powerful tool to study the role of various essential and non-essential elements in cancer is laser ablation-inductively coupled plasma-mass spectrometry (LA-ICP-MS), which allows to map the distributions of trace elements quantitatively with high spatial resolution (typically between 1 and 100 µm) [30]. The simultaneous investigation of protein distributions (e.g., via metal-labelled antibodies) or the parallel investigation via histopathological techniques enables the visualization of the tumor environment and the study of the distribution of elements in the context of specific cancer features and anatomical structures [30, 31]. Different studies have previously investigated the potential of LA-ICP-MS in the context of breast cancer. For example, Riesop et al*.* suggested Zn as a potential biomarker of breast cancer as the histopathological malignancy grade can be directly correlated with Zn concentrations in invasive ductal carcinoma [32]. Gonzalez de Vega et al. showed that levels of Ca, Fe, Cu, and Zn in the tumor area were significantly higher than those found in the non-tumor area, as well as a heterogeneous distribution of the investigated metals in the studied tissue [33]. Similarly, Rusch et al. revealed Zn accumulation in breast cancerous tissue [34]. However, most studies employed scanning mass analyzers (e.g., quadrupoles) which restricted the number of analyzable elements. This is a significant constraint for imaging of tumor and surrounding tissues, which consists of various endogenous elements. This restriction can be overcome by using time-of-flight (ToF) technology for ICP-MS, which enables (quasi-) simultaneous analysis of the entire mass spectra in each recorded pixel. While ICP-ToF–MS has lower duty cycles than quadrupole-based ICP-MS when analyzing only a handful of elements, ICP-ToF–MS becomes advantageous when analyzing increasing numbers of m/z and allows strategies such as post-processing accumulation of isotopes and optimization of the duty cycle to boost sensitivity as shown recently [35]. LA-ICP-ToF–MS has been successfully applied to map endogenous elements in colorectal cancer tissues and to characterize lesions in brain tissues of multiple sclerosis patients [36, 37].

In this work, a quantitative LA-ICP-ToF–MS method was developed to investigate the spatial variations in essential trace and xenobiotic metals in non-metastatic and metastatic breast formalin fixed tissues as well as in healthy controls. Following the identification of interesting correlations between elements and histological stainings, a focus was set on analyzing the quantitative distributions of Fe, Cu, Zn, Sr, and Ba in the cancer tumor niche and stroma.

## Materials and methods

### Sample collection

Paraffin-embedded human breast tissues were provided by Hospital de Jove Foundation in Spain (4 healthy, 7 non-metastatic, and 11 metastatic breast tissues). Hematoxylin and eosin (H&E) staining was used to identify the tumor niche and stroma areas and to characterize the tissue composition. An immunohistochemistry (IHC) procedure was performed for estrogen receptor (ER), human epidermal growth factor receptor 2 (HER2), and progesterone receptor (PR) to characterize the cancer further as stated by ASCO/CAP guidelines [38–40]. Tumors were labelled as positive (1) or negative (0) depending on the presence or absence of these biomarkers. The sample pathological characteristics are listed in Table S1, and the patients were grouped as healthy (H), non-metastatic (NM), and metastatic (M). This study has been approved by Hospital de Jove Foundation Ethics and Investigation Committee (PI02/2018) and follows the national regulations.

### Sample treatment for LA-ICP-ToF-MS

Breast tissue samples were previously fixed in formalin and embedded in paraffin for a better conservation over time. Microtome cuts (Leica Microsystems GmbH, Wetzlar, Germany) were performed to obtain consecutively 5 µm sections which were subsequently placed onto adhesive-coated slides. For deparaffination prior LA-ICP-MS analysis, the tissue slides were heated 60 min in a UF30 plus oven (Memmert) at 60 °C and submerged in different solutions consecutively: 10 min in xylene 100% (Sigma-Aldrich, USA) and then 5 min in 100% ethanol, 95% ethanol, 70% ethanol HPLC grade (CL Chem-Lab, Belgium), and finally in ultrapure water (18.2 MΩ cm, Merck Millipore, Bedford, USA).

It is worth noting that formalin fixation [41], deparaffination, and a washing procedure may have consequences for the distribution and levels of elements throughout all tissue groups. This is a known dilemma as tissues are usually recovered from large (fixed and preserved) libraries, and ideally, future studies should focus on cryo-samples to avoid the potential washout effects. However, the conservation of fresh tissues is more complex as it should be done at − 80ºC to avoid microbiological contamination.

### LA-ICP-ToF-MS bioimaging

LA-ICP-ToF–MS analysis was performed using an Analyte G2 excimer Laser Ablation System (Teledyne Photon Machines, USA) with a wavelength of 193 nm, equipped with an aerosol rapid introduction system (ARIS) and coupled directly to the torch of a Vitesse ICP-ToF–MS platform (Nu Instruments, UK). Instrumental parameters such as laser He flow, nebulizer Ar flow, and torch position were optimized daily to obtain the best sensitivity while ablating a NIST 612 “Trace Elements in Glass” and monitoring the signals for ^115^In and ^238^U. The parameters used for the analysis are summarized in Table S2. The monitored mass range was 20–240 amu while blanking the ranges 24.5–30.5 and 38–47 amu to avoid signal saturation at the detector. Two spectra where binned before baseline correction and 20 after baseline correction and the spectra were stored every 1.03 ms. Elemental mapping was performed using a laser beam spot size of 35 µm (square shape) with a laser dosage of 4. Data acquisition was performed by Nu Codaq software (Nu Instruments).

External calibration was carried out by manufacturing gelatine standards following a protocol previously described by Westerhausen et al*.* [42]. Briefly, 20% pork gelatine (MM ingredients, UK) was heated and mixed with an Amberlyst® 15 ion-exchange resin (Sigma-Aldrich, USA) to reduce the metal background concentrations. Then, the resin was separated by centrifugation, and the clean gelatine was spiked with the desired concentrations of Single Element ICP Standard Solution Roti®Star (Carl Roth, Germany) and filled into molds (Grace Bio-Labs, USA). Calibrations ranged between 0 and 33 µg g^−1^ for Fe and 0 and 19 µg g^−1^ for Cu, Zn, and Sr. To calculate the exact concentrations of the analytes in the standards, an aliquot of each standard was dried overnight, weighed, and digested with 20% nitric acid (CL Chem-Lab Nitric, Belgium). The resulting solution was then diluted with ultrapure water and analyzed using an Agilent 8900 ICP-MS/MS system. The parameters for the cross-quantification are summarized in Table S2, and Fe, Cu, Zn, and Sr calibration curves are displayed in Figure S3. To build the LA-ICP-ToF–MS calibration curves, 5 lines of 200 pixels of each standard were ablated. The average intensities were calculated and plotted against their respective concentration values.

### Data analysis

LA-ICP-ToF–MS data was visualized using Pew^2^ software [43]. The images obtained were previously treated with a rolling median noise treatment (size 5, *k* = 3.0). The background signal due to the glass and adipose tissue was segmented and eliminated using a *k*-means algorithm (*k* = 3, *t* = 1). Then, the signal histogram and median were calculated for sample group comparison and a one-tailed Mann–Whitney *U* test was performed at 95% of confidence to pinpoint significant differences in the metal levels in the three tissue groups (H, NM, M). Concentration medians were expressed with its quartile 1 and 3 (Q1 and Q3) intervals. For the correlation analysis, a Spearman test was used to compare the correlation of the signal of Sr and Ba in the epithelial tissue (moderate < 0.6 and > 0.7, strong > 0.8). Also, for comparison of metal levels in the tumor, stroma as well as in the adipose and the epithelial tissues, a two-tailed Wilcoxon signed-ranked test was performed. *p*-values ≤ 0.05 were considered statistically significant with a 95% of confidence. All statistical data analyses were conducted using Origin Pro 2018.

## Results and discussion

### Image thresholding

**Fig. 1:**
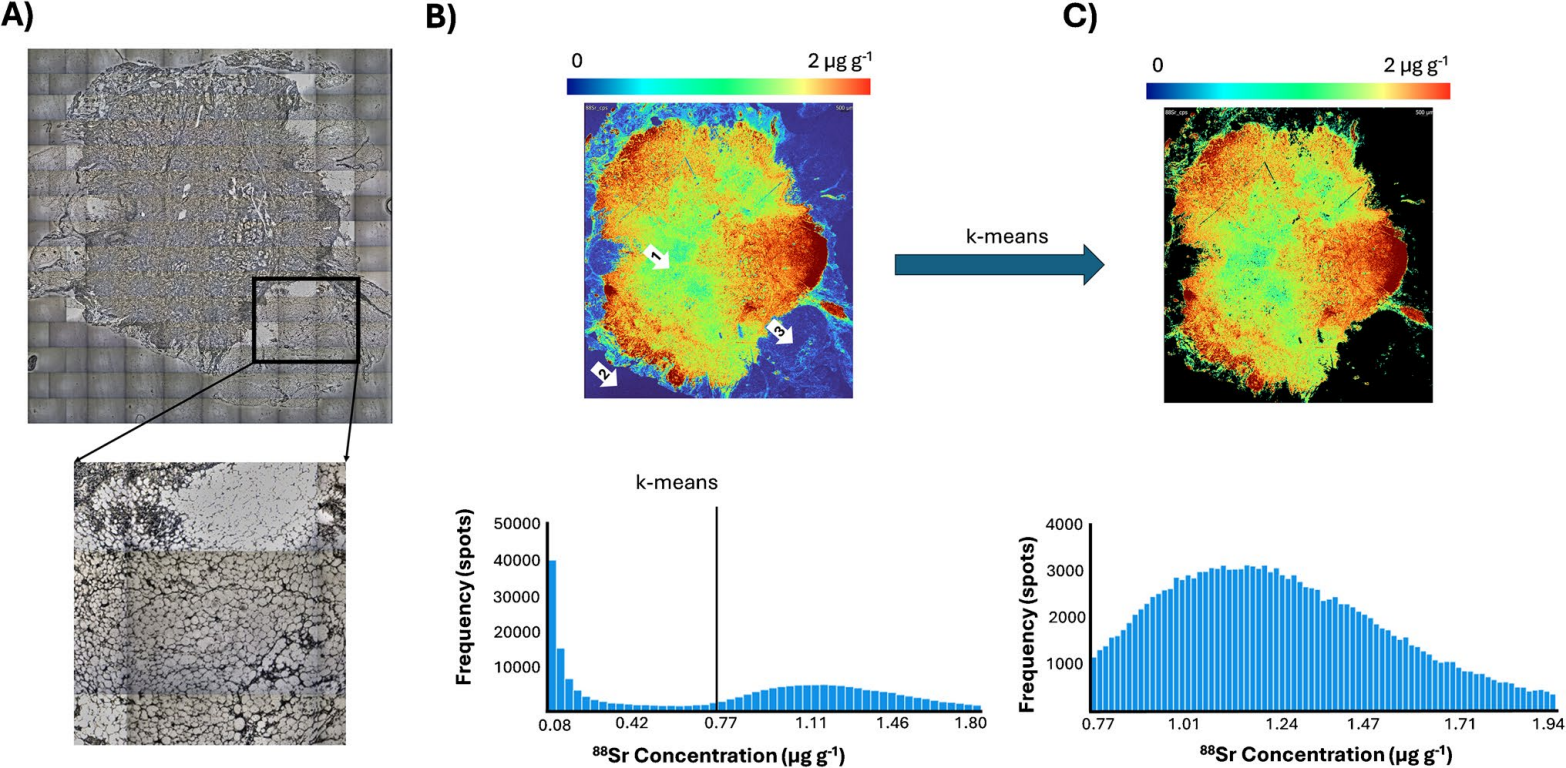
Graphic representation of *k*-means clustering for background and irrelevant tissue removal on a non-metastatic sample. **A** Laser camera image of the analyzed sample with a magnification of the adipose tissue zone. **B** Quantitative biodistribution of ^88^Sr and histogram. **C** Quantitative biodistribution of ^88^Sr and histogram after *k*-means background removal. White arrows 1, 2, and 3 refer to epithelial tissue, glass slide, and adipose tissue, respectively

The complex tissue morphology and cutting process were inherent with some artefacts such as holes, irrelevant tissues (e.g., adipose tissue), and void areas, which need to be disregard when comparing element concentrations [44, 45]. As such, a *k*-means segmentation method was employed to select relevant tissue areas [46]. Following the approach of Castellanos-Garcia et al*.* [47], a *k*-means algorithm with parameters set to *k* = 3 and *t* = 1 was used to differentiate between background, as well as low and high concentration tissue areas. An explanatory graphic of *k*-means operating principle is summarized in Fig. 1. Figure 1A shows a laser camera image of the analyzed sample, including a magnified view of the adipose tissue region. The untreated image is shown in Fig. 1B in which the epithelial tissue (1), glass slide (2), and adipose tissue (3) are shown. These three parts of the untreated image contribute to the median calculations obtaining an underestimation. Using *k*-means for image segmentation allowed to estimate a threshold under which intensities were attributed to irrelevant background and tissue signals. As shown in Fig. 1C, irrelevant areas such as adipose tissues and glass slide areas were masked to not bias median calculations. Adipose tissue removal with *k*-means was validated comparing with the H&E staining and laser camera pictures. When comparing different tissues (adipose and epithelial) or sample regions (tumor niche and stroma), H&E staining was used to identify the tissues/zones. These were then directly compared using their median values, rather than employing *k*-means clustering. It should be noted that the *k*-means algorithm was applied for the analysis of each element individually.

### Quantitative distribution of essential and non-essential elements in breast cancer tissues

**Fig. 2:**
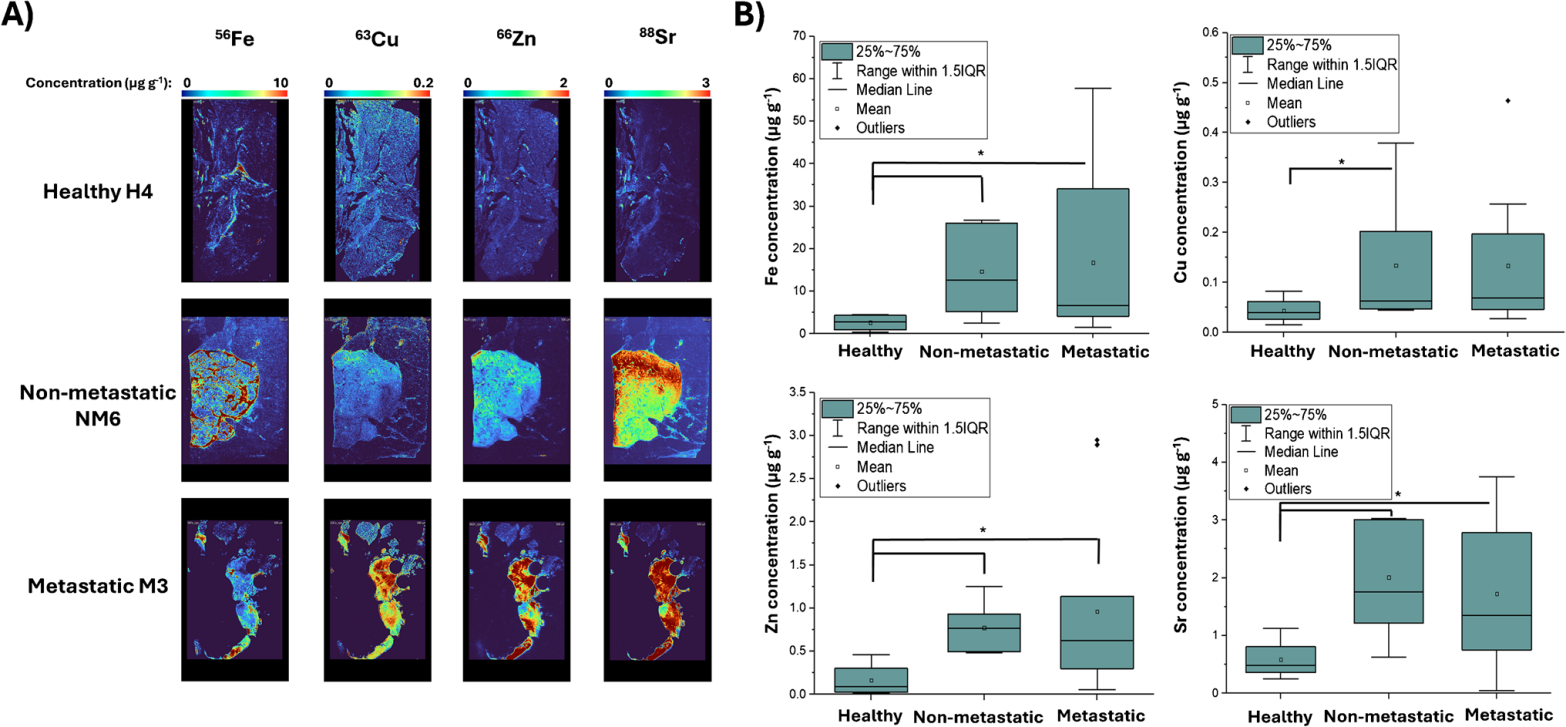
**A** Quantitative distribution for Fe, Cu, Zn, and Sr in the different sample groups. **B** Box chart of the elemental concentrations found in healthy, non-metastatic, and metastatic tissues. Box charts: * represents statistical significance $(p\le \hspace{0.17em}0.05)$ in Mann–Whitney U test

In a first step, figures of merit were determined. Following the ablation of gelatine standards, limits of quantification (LOQs) were calculated as ten times the standard deviation of the blank divided by the calibration curve slope. LOQs were 58 ng g^−1^ for Fe $\left({R}^{2}=0.987\right)$, 16 ng g^−1^ for Cu $({R}^{2}=0.996)$, 83 ng g^−1^ for Zn $({R}^{2}=0.990)$, and 11 ng g^−1^ for Sr $({R}^{2}=0.997)$. Subsequently, quantitative element distributions were determined in tumor and control tissues and compared. Figure 2A presents a comparison of Fe distribution across different tissue groups, revealing a significantly higher Fe concentration in tumoral tissue (epithelial) compared with adipose tissue across all samples. The median Fe concentrations were 0.79 (0.46–1.26) µg g^−1^ in adipose tissue and 4.74 (2.62–13.39) µg g^−1^ in tumoral tissue, with a *p*-value of 9.78 × 10^-5^. A significant increase in Fe was observed when comparing healthy samples with cancer samples (non-metastatic and metastatic) with concentrations of 2.9 (0.9–4.4) µg g^−1^ for healthy, 12.6 (5.2–26.1) µg g^−1^ for non-metastatic (*p* = 0.015), and 6.6 (4.1–34.1) µg g^−1^ for metastatic (*p* = 0.022) (see Fig. 2B). However, no significant differences were observed between both cancer groups (*p* = 0.607). An increase in signal was observed in some metastatic samples due to the presence of hot spots in different parts of the tissues. In some samples (3/22), the H&E staining identified certain Fe hot spots as breast ducts. However, as not all the sample cuts presented ducts, this correlation could not be studied further. Certain areas of high concentration warrant further investigation to determine their potential correlation with the cancer progression and/or metastatic process.

As shown in Fig. 2A, Cu was relatively heterogeneous distributed showing variations between adipose and epithelial tissue with increasing concentration in the latter, with 0.03 (0.01–0.06) µg g^−1^ and 0.05 (0.03–0.21) µg g^−1^, respectively (*p* = 4.30 × 10^−5^). When patient groups were compared, a similar behavior to Fe was found. There was a noticeable increase in Cu concentration from healthy to cancer samples, with median levels of 0.04 (0.03–0.06) µg g^−1^ and 0.07 (0.05–0.20) µg g^−1^, respectively (*p* = 0.003). Significant differences were also found between non-metastatic (0.06 (0.05–0.20) µg g^−1^) and healthy groups (*p* = 0.036). However, despite an increase, the difference between healthy and metastatic group (0.07 (0.05–0.20) µg g^−1^) was not statistically significant (*p* = 0.066) (see Fig. 2B).

Similar variability in distributions between adipose and epithelial tissue was found for Zn, with a higher concentration of Zn in the epithelial tissue (Fig. 2A). The calculated median concentrations were 0.06 (0.02–0.19) µg g^−1^ for adipose tissue and 0.44 (0.14–7.48) µg g^−1^ for epithelial tissue with *p* = 4.30 × 10^−5^. Zn levels were significantly lower in the healthy group 0.12 (0.08–0.30) µg g^−1^ compared with the cancer groups: 0.78 (0.50–0.93) µg g^−1^ for non-metastatic (*p* = 0.005) and 0.63 (0.30–1.14) for metastatic (*p* = 0.038). However, no significant differences were observed between the non-metastatic and metastatic cancer groups (*p* = 0.737) (see Fig. 2B).

It should be also highlighted that the samples with the highest Zn concentration are different than the ones for Fe and Cu (Table S1). This means that although the cancer groups follow a similar pattern, the samples with the higher amount of Fe, Cu, or Zn are different. This could be attributed to the competition of metal ions for metalloprotein cofactor binding sites. It is well established that tumor cell metabolism is altered, affecting the binding and storage of metal ions such as Fe, Cu, and Zn. For instance, proteins like metallothioneins (MTs), solute carrier family 31 member 1 (CTR1) that transports Cu, transferrin receptors for Fe, and various Zn transporters are overexpressed in several types of cancer [48–50].

Sr is a ubiquitous element commonly incorporated into biological structures due to its similarity to Ca; however, its impact and metabolic roles are vastly unknown. Significant differences in Sr levels were observed between adipose and epithelial tissues, with the Sr distribution in epithelial tissue being notably more homogeneous compared with other previously analyzed elements. The calculated median concentrations were 0.13 (0.08–0.18) µg g^−1^ for adipose tissue and 1.7 (0.79–3.11) µg g^−1^ for epithelial tissue showing a significant difference (*p* = 4.30 × 10^−5^) (Fig. 2A). Sr also exhibits the largest concentration differences between sample groups as shown in Fig. 2B. When comparing healthy and cancer samples, the tumoral Sr concentration is three times higher than the control group. Concentrations were 0.5 (0.4–0.8) µg g^−1^ for healthy, 1.8 (1.2–3.0) µg g^−1^ for non-metastatic (*p* = 0.009), and 1.4 (0.8–2.8) µg g^−1^ for metastatic (*p* = 0.022). However, similar to the previously studied elements, no significant differences were observed between the M and NM groups (*p* = 0.793). This study does not infer why Sr could be accumulated in the tumoral tissue. However, metabolic activity in cancer cells is increased, and elemental selectivity is reduced which suggests that Sr may be transported and accumulated through Ca^2+^ channels [51].

LA-ICP-ToF-MS revealed a diverse distribution of Fe, Cu, Zn, and Sr within breast tissue, and variations in the concentrations of these elements were found across different tissue regions and tissue groups. The spatial distribution by tissue zones (identified by H&E and laser camera images) indicated a higher accumulation of the analyzed metals in the epithelial tissue compared with adipose tissue, likely due to the predominant accumulation of lipids in adipocytes [52] which in consequence decreases metal accumulation. However, investigated elements were found significantly increased in cancer samples relative to the healthy group. This could be explained by the accelerated metabolism of the tumor cells and their non-selective elemental uptake [51]. These findings are in line with previous studies on breast cancer [53] and other types of cancer [54, 55], in which serum or digested tissues were analyzed by stand-alone ICP-MS. In these studies, increased concentrations in cancer affected tissues of Fe, Cu, and Zn among other elements like Ca, manganese (Mn), Hg, Pb, and Se were shown. However, it is worth pointing out that these studies did not achieve the spatial resolution of element distribution, which becomes possible when employing LA-ICP-MS.

### Sr and Ba correlation

**Fig. 3:**
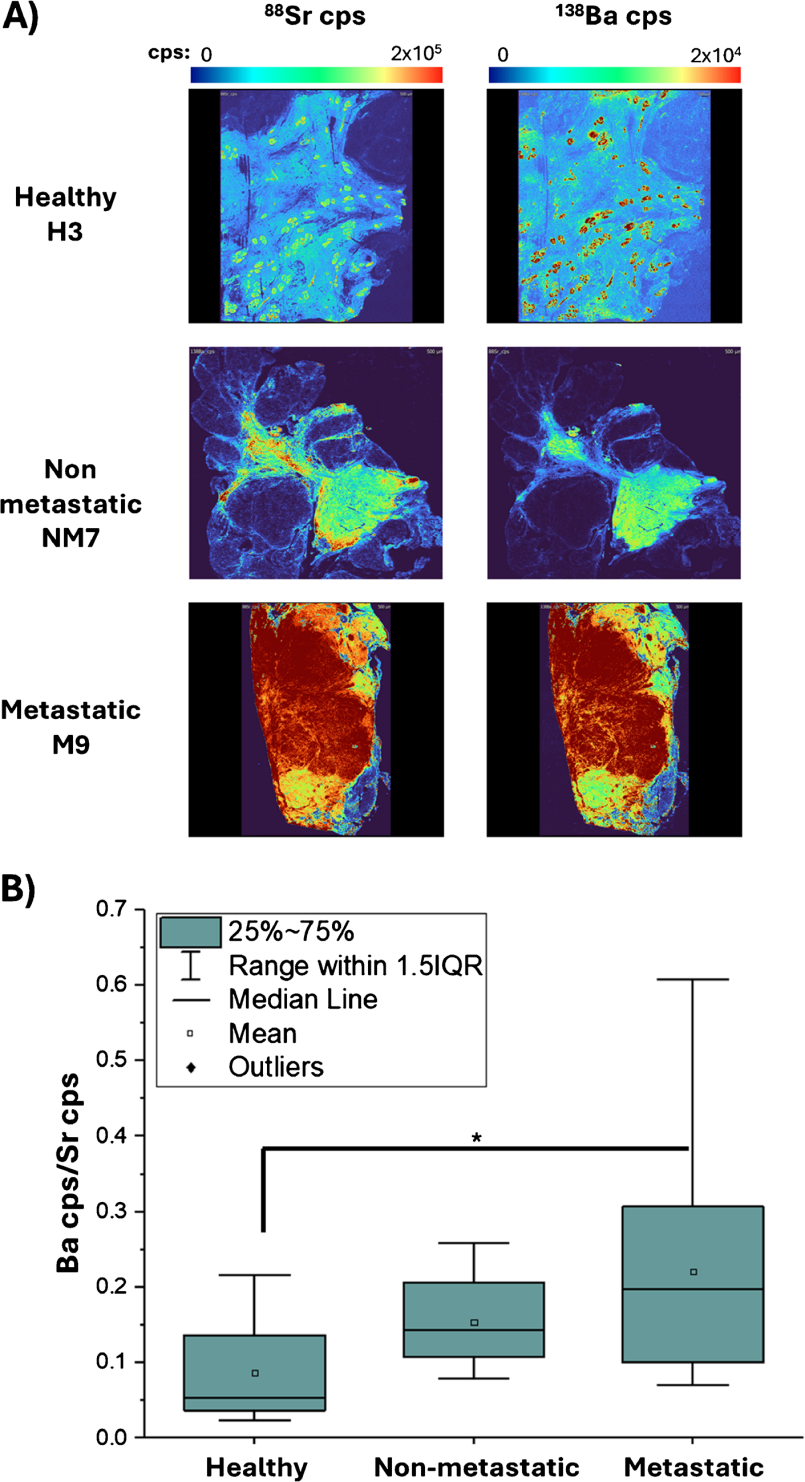
**A** Qualitative elemental distribution (cps) of Sr and Ba in the studied groups (healthy, non-metastatic, metastatic). **B** Box plots representing the Ba/Sr ratio for each studied group. The Ba signal was normalized using the Sr signal from a gelatine standard of 1.31 µg g^-1^. Box plots:* represents statistical significance (*p* ≤ 0.05) in a Mann–Whitney U test

In recent years, several studies focused on the examination of heavy metals exposure and subsequent metabolic pathways in the context of breast cancer [56, 57]. While the effects of heavy metals such as lead, chromium, nickel, cadmium, cobalt, and mercury were previously investigated, the role of other heavy metal entities is still obscured [56, 58–60]. In this study, a ToF-analyzer for LA-ICP-MS was used, which provides information on (almost) any element across the periodic table in any recorded mass spectrum and pixel. As such, LA-ICP-ToF–MS is predestined to examine the accumulation of various heavy metals in breast cancer tissues to reveal new correlations and to interrogate the role, levels, and/or effects of certain entities. In this study, LA-ICP-ToF–MS revealed high levels of Ba in epithelial tissue. It is not known how Ba is taken up and accumulates into cancer areas, but due to the similar chemistry of Sr and Ca, it appears reasonable that Ba^2+^ is taken up via Ca^2+^ channels of cancer cells, which exhibit a lower selectivity for specific ionic species. Ba has no functional role in the human body, and it is likely that exposure to Ba is mainly caused by dietary factors [61]. It is unknown how elements such as Sr and Ba are implicated in breast cancer and whether pathophysiology is somewhat affected. However, their accumulation in cancer tissues is still interesting and may qualify them as bio-indicative markers. When comparing the qualitive distribution of Sr and Ba, a high/moderate colocalization between the two elements can be observed (see Fig. 3A**)**. Sr and Ba were very similarly distributed in all three sample groups, and their correlation and colocalization was quantified using a Spearman correlation test. The correlation coefficients revealed a moderate to strong (moderate < 0.6 and > 0.7, high > 0.8) [62] correlation in 16/22 samples (from 0.46 to 0.90) indicating a solid association between Sr and Ba suggesting that similar quantitative differences could be expected for Ba as well. Finally, to estimate the variation of Ba and its spatiotemporal correlation to Sr across the investigated tissue groups, the Ba signal was normalized with a Sr-spiked gelatine standard. Sr and Ba exhibit similar behavior and distribution across the tissue, with Sr being relatively homogeneously distributed in the adipose tissue. This normalization, represented in Fig. 3B showed a significant increase of Ba levels in metastatic samples (0.20 (0.10–0.31) µg g^−1^) compared against healthy samples (0.05 (0.04–0.14) µg g^−1^) with *p* = 0.029. It should be noted that all studies were done with a sample group size of 11 tissues or less, suggesting that statistical differences could be improved with a larger sample size.

### Differences between tumor niche and stroma

**Fig. 4:**
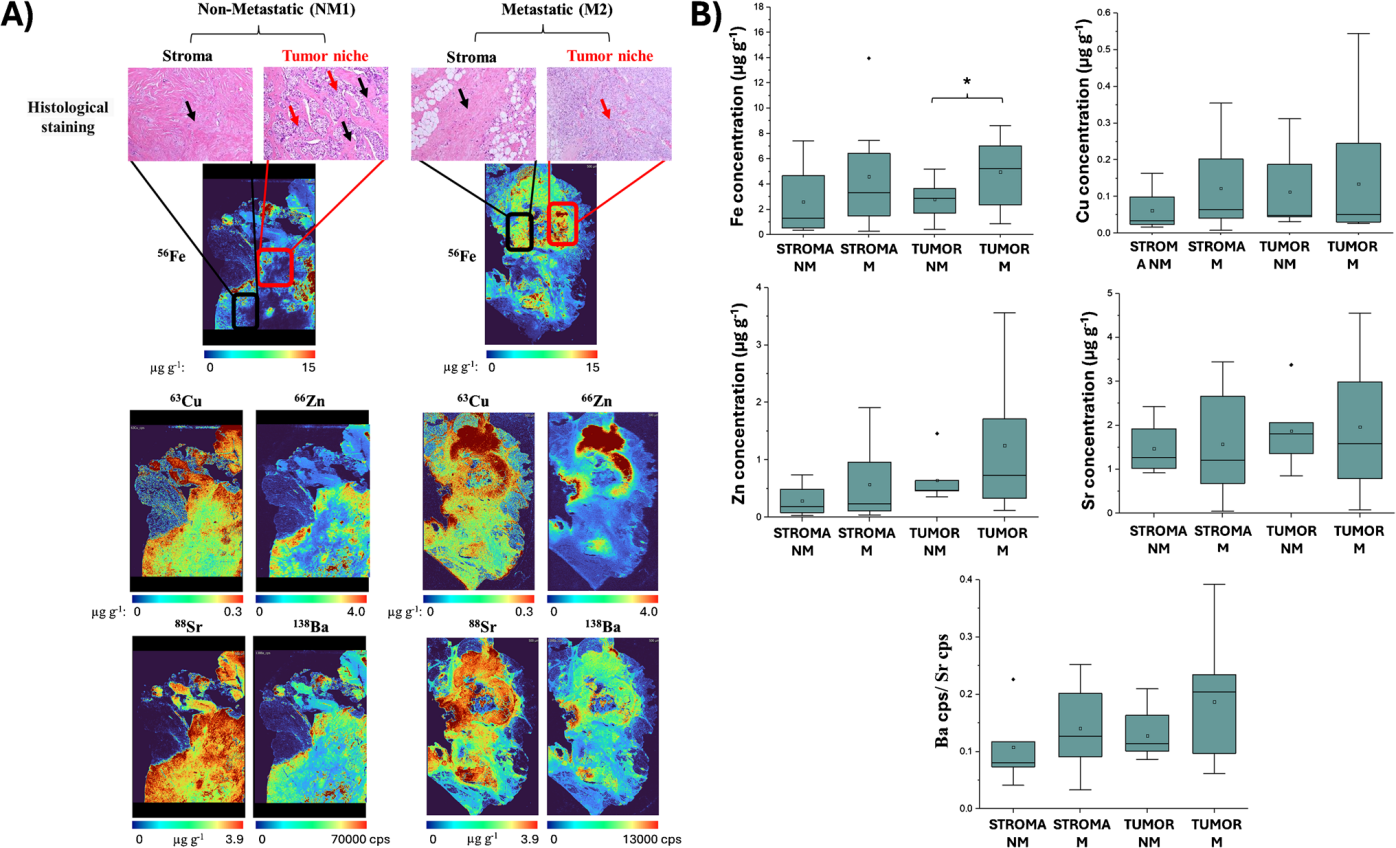
Comparison of the tumor niche and stroma distributions in the different sample groups. **A** Tumor niche and stroma as observed in H&E staining, along with quantitative elemental distributions of Fe, Cu, Zn, and Sr and qualitative distribution of Ba. **B** Concentration results comparing Fe, Cu, Zn, Sr, and Ba (the latter normalized using a Sr standard) across the different sample groups. Black arrows in the H&E image represent the stroma and red arrows the tumor niche. Box plots: * represents statistical significance (*p* ≤ 0.05) in a Mann–Whitney U test

To gain a better understanding of the elemental distribution in the BC tissues analyzed in this study, both the tumor niche, composed solely of tumor cells, and the stroma, which includes non-tumor cells like fibroblasts and immune cells, were localized with an H&E staining. It is worth noting that in some samples, the cancer was so widespread that there was no stroma area. This decreased the sample number to 5 non-metastatic and 8 metastatic samples for the stroma analysis. Some studies have focused on the expression of protein biomarkers in these two areas [29, 63]; however, in this study, we suggest interrogating the levels of both essential and non-essential elements as biomarkers and propose to study their quantitative distribution in stroma and tumor niche areas to enable differentiation and to assess different tissue zones separately. A comparison between the tumor niche and stroma of all investigated elements is summarized in Fig. 4A. The study of the microenvironment and tissue differentiation was conducted using H&E staining, as shown above the elemental maps. In the stroma areas, connective tissue with small nuclei and some adipose cells in the metastatic sample were observed. Meanwhile for the tumor area, apart from some voids and a few adipose cells in the metastatic sample, a high number of tumor cells were identified by its big nuclei.

Cu, Zn, and Sr levels were significantly higher in the tumor area compared with the stroma, with *p*-values of 0.021, 0.003, and 0.011, respectively. In contrast, Fe did not show significant differences, likely due to its more heterogeneous distribution within the tissue (*p* = 0.246). The comparison between non-metastatic and metastatic samples revealed increased variability among patients in the metastatic group, indicating greater heterogeneity in both tumor and stroma areas, as illustrated in Fig. 4B. Specifically for Fe, there was a significant increase in concentration in tumor areas of cancer patients, with levels of 2.87 (1.71–3.65) µg g^−1^ for non-metastatic and 5.22 (2.37–7.02) µg g^−1^ for metastatic (*p* = 0.049). Although Cu and Zn displayed a similar trend, no significant differences were found between cancer groups. For Sr, while variability was higher in metastatic samples, no significant differences were observed, and distinct distribution patterns were evident. It is important to note that these differences should be further investigated using fresh samples to avoid potential metal washout during the deparaffinization process.

## Conclusions

The elemental composition of BC tissues was assessed via LA-ICP-ToF–MS enabling multi-element detection in each recorded pixel. This approach allowed the detection of little studied metals (Sr and Ba), which may have utility to be considered for detecting cancer areas and tumor segmentation. One possible explanation for the increase in Sr and Ba could be an enhanced and unselective cancer metabolism.

For quantification, low background gelatine standards were prepared. The LOQs for the investigated metals were between 11 and 83 ng g^−1^. All studied elements were found more concentrated in the epithelial tissue than the adipose tissue, probably due to the mainly storage of lipids by the adipose tissue which means that metals are not accumulated there. Quantitative analysis of Cu, Fe, Zn, and Sr showed a significant increase of these elements in cancer samples; nevertheless, no differences between the non-metastatic and metastatic groups were found. All these elements were overexpressed in cancer, and its accumulation seemed independent of the cancer stage or metastatic process. This may indicate that metal accumulation is likely occurring during the early stages of cancer and not significantly enhanced during later progression stages.

Parallel histology staining could furthermore help distinguish the tumor niche and stroma, allowing a more precise and detailed study of the tumor areas. All elements, except Fe, were found to be elevated in the tumor compared to the surrounding stroma, likely due to the accelerated metabolism of cancer cells. When comparing non-metastatic and metastatic samples, Fe was significantly more concentrated in the tumor areas of metastatic samples compared with non-metastatic samples. Although Cu and Zn showed similar trends, no significant differences were observed between cancer groups. It is important to recognize that this study involved only 7 non-metastatic and 11 metastatic samples, so a larger sample size may be required to confirm the findings and reduce variability. Future research should aim to include a greater number of specimens and ideally focus on fresh-frozen tissues to minimize potential bias introduced during sample preparation.

## Supplementary Information

Below is the link to the electronic supplementary material.

Supplementary file1 (DOCX 141 KB)

## Data Availability

The data supporting this article have been included in the main manuscript and in the Supplementary Information.
