# Supplementary material for: Quantitative distribution of essential elements and non-essential metals in breast cancer tissues by LA-ICP-TOF–MS
Source: Anal Bioanal Chem. 2024 Nov 18;417(2):361–71. doi: 10.1007/s00216-024-05652-8 (PMC11698889; doi:10.1007/s00216-024-05652-8)

**Quantitative distribution of essential elements and non-essential metals in breast cancer tissues by LA-ICP-TOF-MS**

Sara Escudero-Cernuda^1^, David Clases^2^, Noemi Eiro^3^, Luis O. González^3^, María Fraile^3^, Francisco J. Vizoso^3^, María Luisa Fernández-Sánchez^1*^, Raquel González de Vega^2*^.

^1^Department of Physical and Analytical Chemistry, University of Oviedo, Oviedo, Spain.

^2^Institute of Chemistry, University of Graz, Graz, Austria.

^3^Research Unit, Jove Hospital Foundation, Gijón, Spain.

***Corresponding authors:** [marisafs@uniovi.es](mailto:marisafs@uniovi.es) , [raquel.gonzalez-de-vega@uni-graz.at](mailto:raquel.gonzalez-de-vega@uni-graz.at)

**Table of contents:**

Table S1: Clinicopathological features for the different breast tissues sorted by clinical group and quantitative LA-ICP-MS results

Table S2: Laser ablation and ICP-MS instrumental parameters

Figure S1: Fe, Cu, Zn and Sr exemplary calibration curves

**Table S1.** Clinicopathological features for the different breast tissues sorted by clinical group and metal LA-ICP-MS quantification results. Tumour size: (T1) less than 2 cm, (T2) between 2 cm and 5 cm. Histological grade: (3) little differentiated; (1) well differentiated; (2) moderately and (3) little differentiated. For IHC biomarkers (ER, PR and HER2) determination: Positive = 1, Negative =0. Lymph nodes nomenclature: Non affected =0, affected =1. Native element concentration for Fe, Cu, Zn and Sr expressed by their median (µg g^-1^).

| Sample ID | Sample Group | ER | PR | HER2 | Tumour Size | Stage | Histological Grade | Lymph Node | ^56^Fe (µg g^-1^) | ^63^Cu (µg g^-1^) | ^66^Zn (µg g^-1^) | ^88^Sr (µg g^-1^) |  |
| --- | --- | --- | --- | --- | --- | --- | --- | --- | --- | --- | --- | --- | --- |
| H1 | Healthy |  |  |  |  |  |  |  | 0.359 | 0.016 | 0.083* | 0.255 |  |
| H2 | Healthy |  |  |  |  |  |  |  | 4.278 | 0.038 | 0.083* | 0.466 |  |
| H3 | Healthy |  |  |  |  |  |  |  | 1.448 | 0.083 | 0.147 | 1.122 |  |
| H4 | Healthy |  |  |  |  |  |  |  | 4.549 | 0.042 | 0.463 | 0.255 |  |
| NM1 | | Non-metastatic | 1 | 1 | 0 | T1 | 1 | 1 | 0 | 26.070 | 0.203 | 1.249 | 3.028 |
| NM2 | | Non-metastatic | 1 | 1 | 0 | T2 | 2 | 1 | 1 | 9.151 | 0.048 | 0.499 | 1.220 |
| NM3 | | Non-metastatic | 0 | 0 | 1 | T1 | 2 | 3 | 1 | 26.780 | 0.063 | 0.929 | 0.631 |
| NM4 | | Non-metastatic | 1 | 1 | 0 | T1 | 2 | 3 | 1 | 2.513 | 0.379 | 0.771 | 3.007 |
| NM5 | | Non-metastatic | 1 | 1 | 0 | T1 | 1 | 1 | 0 | 5.171 | 0.059 | 0.591 | 1.691 |
| NM6 | | Non-metastatic | 0 | 0 | 0 | T2 | 2 | 3 | 1 | 20.630 | 0.045 | 0.483 | 1.757 |
| NM7 | | Non-metastatic | 0 | 0 | 1 | T2 | 2 | 3 | 1 | 12.620 | 0.146 | 0.897 | 2.749 |
| M1 | | Metastatic | 1 | 1 | 0 | T1 | 1 | 1 | 0 | 6.613 | 0.035 | 0.333 | 0.752 |
| M2 | | Metastatic | 1 | 1 | 1 | T1 | 1 | 2 | 0 | 6.624 | 0.197 | 2.947 | 2.398 |
| M3 | | Metastatic | 0 | 0 | 0 | T2 | 2 | 3 | 0 | 4.093 | 0.106 | 0.627 | 2.785 |
| M4 | | Metastatic | 1 | 1 | 0 | T1 | 2 | 3 | 1 | 17.820 | 0.467 | 2.895 | 3.747 |
| M5 | | Metastatic | 1 | 1 | 1 | T2 | 2 | 3 | 0 | 10.79 | 0.069 | 0.725 | 1.353 |
| M6 | | Metastatic | 1 | 1 | 1 | T1 | 1 | 1 | 0 | 36.700 | 0.059 | 0.325 | 1.348 |
| M7 | | Metastatic | 1 | 1 | 0 | T2 | 2 | 2 | 1 | 5.403 | 0.257 | 1.139 | 3.005 |
| M8 | | Metastatic | 1 | 1 | 0 | T2 | 2 | 2 | 1 | 34.070 | 0.027 | 1.139 | 0.045 |
| M9 | | Metastatic | 0 | 0 | 0 | T2 | 2 | 3 | 0 | 1.553 | 0.150 | 0.083* | 1.291 |
| M10 | | Metastatic | 1 | 1 | 0 | T2 | 3 | 2 | 1 | 2.987 | 0.063 | 0.299 | 0.629 |
| M11 | | Metastatic | 0 | 0 | 0 | T1 | 1 | 3 | 1 | 57.780 | 0.046 | 1.126 | 1.635 |

*< Zinc’s LOQ = 0.083 µg g^-1^

**Table S2.** Laser ablation and ICP instrumental parameters for the elemental quantification in BC tissues**.**

| **Technique** | **Parameter** | **Value** |
| --- | --- | --- |
| **Laser Ablation** | Spot size (µm) | 35 |
|  | Repetition frequency (Hz) | 100 |
|  | Fluence (J/cm^2^) | 1 |
|  | Dosage | 4 |
|  | Speed (µm s^-1^) | 875 |
|  | He flow (L min^-1^) | 0.5 – 0.65 |
| **ICP-TOF-MS** | Radiofrequency power (kW) | 1.3 |
|  | Carrier gas flow (L min^-1^) | 1.10 – 1.35 |
|  | Collision cell gas | He and H_2_ |
|  | He flow (mL min ^-1^) | 18 |
|  | H_2_ flow (mL min ^-1^) | 12 |
| **ICP-MS/MS** | Radiofrequency power (W) | 1600 |
|  | Sampling depth (mm) | 6.7 |
|  | Carrier gas flow (L min ^-1^) | 1.19 |
|  | Collission cell gas | He |
|  | He flow (mL min^-1^) | 5 |

**Figure S1**. External calibration with gelatine-mold standards. A) Exemplary intensity profile along an ablated line of the highest concentration gelatine standard. B) Fe, Cu, Zn and Sr calibration curves. Calibration curves were constructed using the average intensity profiles of five lines.


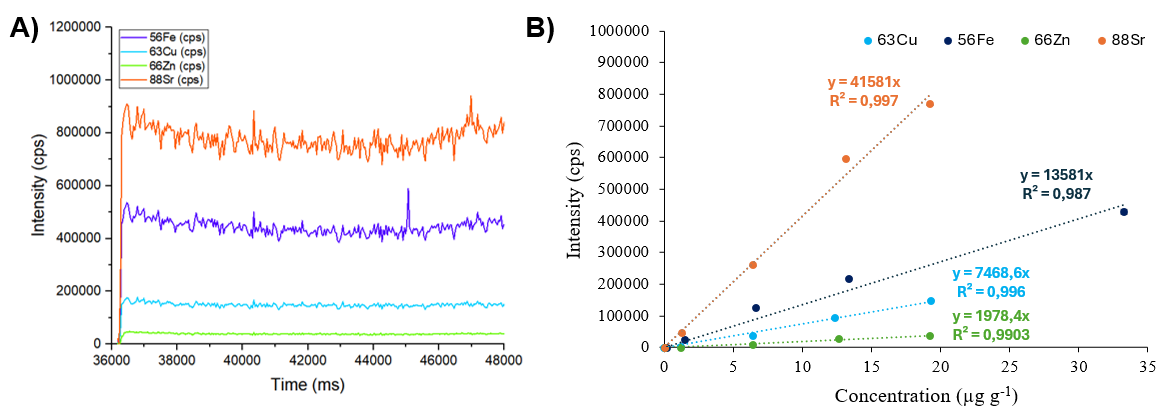

Supplement: Supplementary file 1 — Supplementary file1 (DOCX 141 KB) [file 216_2024_5652_MOESM1_ESM.docx]
